# Computer-aided diagnosis system for thoracic computed tomography of rib fractures in older emergency patients: A preliminary study

**DOI:** 10.1371/journal.pone.0351988

**Published:** 2026-06-17

**Authors:** Shan Xiong, Wenze Wu, Sibin Liu, Jianmin Cheng, Bing Wan

**Affiliations:** 1 Department of Radiology, Jingzhou Hospital Affiliated to Yangtze University, Jingzhou, China‌‌; 2 Department of Radiology, The Second Affiliated Hospital and Yuying Children’s Hospital of Wenzhou Medical University, Wenzhou, China; 3 Department of Radiology, Affiliated Renhe Hospital of China Three Gorges University, Yichang, China; Indiana University School of Medicine, UNITED STATES OF AMERICA

## Abstract

The peculiarities of older individuals related to osteoporosis and hyperostosis may lead to a higher rate of misdiagnosis of rib fractures on computed tomography (CT) images in older than in middle-aged/young people when using radiologist-only reading. However, none of these studies on rib fracture computer-aided diagnostic (CAD) systems grouped patients by age or evaluated the value of using CAD in older patients. To address these gaps, we divided 1,012 blunt chest trauma emergency patients who underwent chest CT into middle-aged/young and older groups with a cutoff age of 60 years. CT images were read by six radiologists from three institutions (each with 7 years of experience in thoracic CT diagnosis) using two reading methods, radiologist-only and radiologist-CAD reading, to explore the value of a deep learning (DL)-based CAD system for detecting rib fractures in emergency older patients. The final findings of the independent panel consisting of two senior radiologists with or without a thoracic surgeon were set as the reference standard. The sensitivity was calculated by dividing the number of true positives by the overall number of fractures, as confirmed by an expert panel. The false positives per patient (FPPP) was calculated by dividing the number of false positives by the overall number of patients. Sensitivity and FPPP were used to evaluate the diagnostic efficiency. Sensitivity, FPPP, and reading time were compared between the two groups, as well as reading methods. The results showed the following: (1) Sensitivity for detecting fresh fractures using radiologist-only reading was lower in the older than in the middle-aged/young group (86.7% [95% confidence interval (CI): 86.3%, 91.7%] vs. 91.5% [95% CI: 89.9%, 92.9%], *p* < 0.05). With the assistance of the CAD system, the sensitivity increased in the older group to the same level as that in the middle-aged/young group using radiologist-only reading (92.5% [95% CI: 90.4%, 94.2%] vs. 91.5% [95% CI: 89.9%, 92.9%], *p* > 0.05). (2) The FPPP of fresh fractures with radiologist-only reading was higher in the older than in the middle-aged/young group (0.47 vs. 0.37, *p* < 0.05). With the assistance of the CAD system, the FPPP in the older group decreased to the same level as that in the middle-aged/young group when using radiologist-only or radiologist-CAD reading (0.37 vs. 0.37/0.39, *p* > 0.05). (3) The reading time of fresh fractures when using radiologist-only reading was longer in the older than in the middle-aged/young group (6.1 vs 5.4 min, *p* < 0.05). With the assistance of the CAD system, the reading time in the older group was reduced by approximately 36% (*p* < 0.05). We conclude that the efficiency of intermediate-level radiologists in diagnosing fresh rib fractures by radiologist-only reading in older emergency patients was lower than that in middle-aged/young patients. When a DL-based CAD system assists radiologists, the diagnostic efficiency of identifying fresh fractures in older patients improves to the same level as independent radiologist-only reading in middle-aged/young patients while reducing the reading time.

## Introduction

Rib fractures commonly occur in patients with blunt chest trauma [1]. While minor fractures have minimal health consequences, rib fractures can be life-threatening as the number of fractures increases and complications worsen [2]. Hence, accurate diagnosis of patients with rib fractures is essential. Multirow spiral thin-slice computed tomography (CT) with a slice thickness of < 2 mm is now the most sensitive method for detecting rib fractures [3]. Nevertheless, rib-by-rib and side-by-side comparisons of many thin-slice images obtained during the diagnostic process are time consuming and cumbersome for radiologists [3,4]. Moreover, the number of radiologists does not keep pace with the required examinations [5].

Furthermore, CT-based rib fracture diagnosis in older emergency patients has certain limitations. The prevalence of osteoporosis is higher in older than middle-aged/young people [6–8]. The decrease in bone density due to osteoporosis [9–12] may cause thinning of the rib cortex and reduction of bone trabeculae in CT images [9, 13–16], which may interfere with the radiologist’s observation of a lucent fracture line in the images. In contrast, the proportion of hyperostosis [17,18] is higher in older people than in middle-aged/young people. This finding primarily reflects degenerative spondylotic changes, where loss of disc height and altered biomechanics at the costovertebral junctions stimulate reactive subperiosteal bone formation to compensate for joint instability [19]. Decades of accumulated mechanical stress on these articulations further promotes aberrant ossification, leading to cortical thickening and irregularity [20]. Such rib cortical thickening and irregular morphology on CT images may interfere with radiologists’ diagnosis of fractures. These peculiarities may lead to a higher rate of misdiagnosis (including false negatives and positives) of rib fractures in older than in middle-aged/young people when using radiologist-only reading. Additionally, the inaccurate diagnosis of rib fractures may lead to legal disputes [21,22]. These issues may overwhelm radiologists who diagnose rib fractures using manual reading alone in older patients.

In recent years, several studies on computer-aided diagnostic (CAD) systems for rib fracture diagnosis based on deep learning (DL) have been published [23–31], as these systems can improve rib fracture diagnostic efficiency [32,33]. For example, some studies have reported [23] the use of a DL algorithm for detecting rib fractures, which achieved a sensitivity of 87.4%, with 0.16 false positives per examination in approximately 500 patients, suggesting that the potential clinical value of the DL algorithm may be of value in assisting radiologists in screening fractures and reducing missed diagnoses. Jin et al. [24] developed a DL model for rib fracture detection. They obtained a sensitivity of 92.9% and false positives per scan of 5.27 in a test cohort that included 900 patients, suggesting that the model would help radiologists achieve a higher sensitivity than that achieved by manual reading alone. Tan et al. [31] found that the sensitivity of rib fracture diagnosis by attending radiologists assisted by DL-based CAD increased from 94.56% to 95.67% compared with radiologist reading alone.

However, none of these studies on rib fracture CAD systems grouped patients by age or evaluated the value of using CAD in older patients. In addition to the peculiarities of older individuals related to osteoporosis and hyperostosis, some radiologists, especially intermediate-level radiologists who have recently assumed responsibility for reviewing reports in our institution, report that they have less confidence in CT-based rib fracture diagnosis in older individuals than in middle-aged/young people. Therefore, we hypothesized that the diagnostic efficiency of radiologists with a certain level of experience in performing independent reading of CT images for rib fractures in older individuals would be lower than that in middle-aged/young people. This study used sensitivity at the lesion level and false positives per patient (FPPP) to evaluate diagnostic efficiency. We first assessed the difference in diagnostic efficiency between two different age groups with a cutoff age of 60 years read independently by radiologists with 7 years of experience; second, we applied a DL-based CAD system to diagnosis in two different age groups to assess its application value in emergency older patients, if a difference in diagnostic efficiency was found between the two different age groups.

## Materials and methods

### Ethics statement

All procedures involving human participants were performed per the ethical standards of the Institutional Research Committee and the 1964 Helsinki Declaration. The authors/radiologists did not have access to information that could identify the individual participants during or after data collection. The Institutional Review Board of Jingzhou Hospital Affiliated to Yangtze University approved the study, and the need for informed consent was waived owing to its retrospective nature.

### Dataset and classification criteria

**Fig 1:**
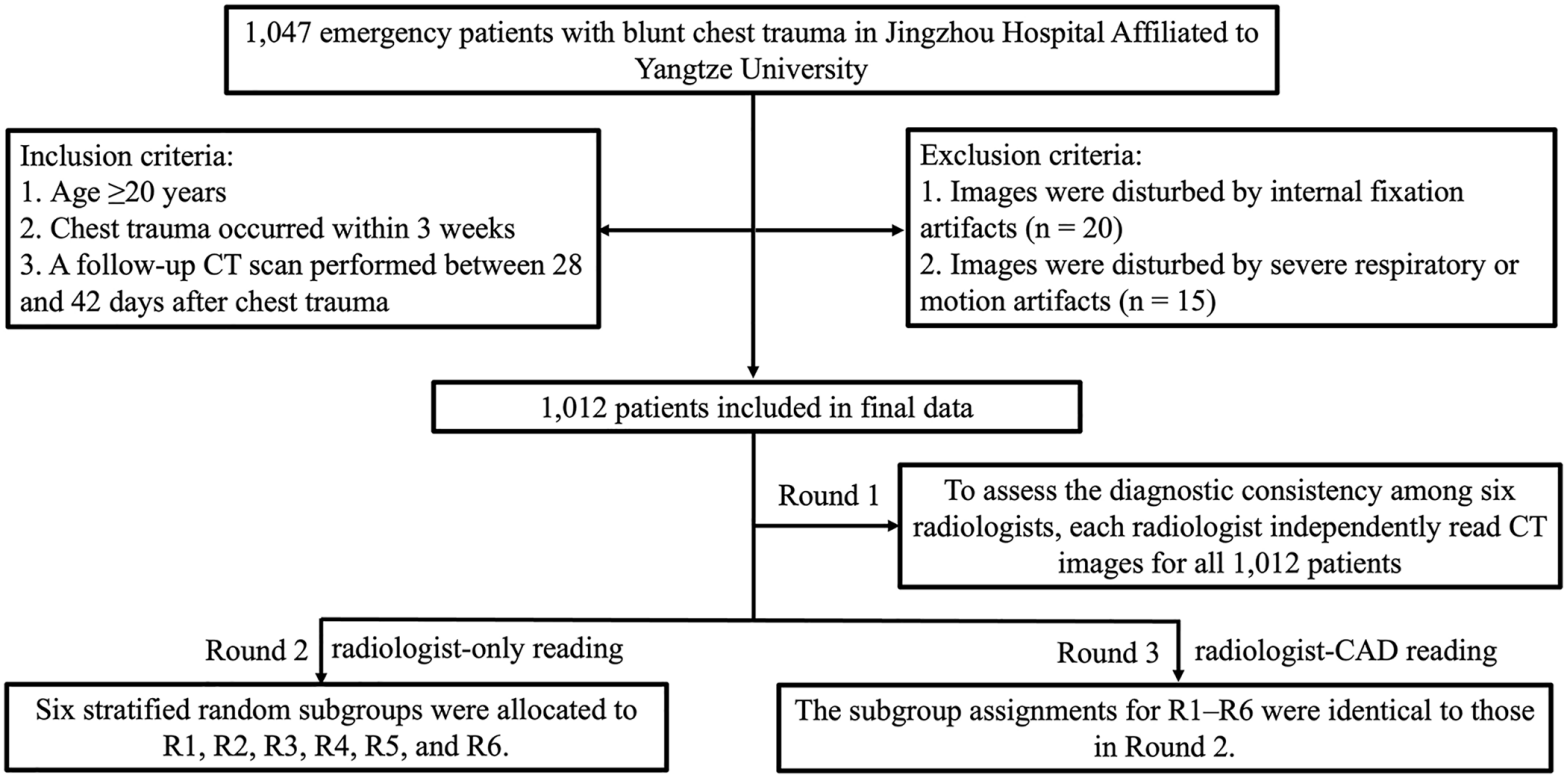
The flow of rib fracture diagnosis in different age groups using different reading methods. R1, R2, R3, R4, R5, and R6 represent radiologists 1, 2, 3, 4, 5, and 6, respectively.

Clinical and imaging data from 1,047 blunt chest trauma were selected using a picture archiving and communication system. These patients underwent emergency chest CT scans at Jingzhou Hospital Affiliated to Yangtze University, between January 2022 and December 2022. The inclusion criteria were as follows: age ≥ 20 years, chest trauma that occurred within 3 weeks, and a follow-up CT scan performed between 28 and 42 days (median time, 31 days) after chest trauma. The exclusion criteria were as follows: images disturbed by internal fixation artifacts (n = 20) or severe respiratory or motion artifacts (n = 15). Finally, 1,012 patients were included in the analysis (Table 1). The diagnoses of rib fractures in different age groups using different reading methods are summarized in Fig 1.

**Table 1:** Patient demographics.

	Group 1	Group 2
**Number**	668	344
**Age (years)**	38.9 ± 10.1 (20–59)	70.7 ± 7.6 (60–87)
**Male/Female**	422/246	213/131

**Note:** Group 1 and Group 2 represent the middle-aged/young group and the older group, respectively.

In this study, we classified fresh fractures as either complete or incomplete. A fresh fracture was defined as a fracture occurring within 3 weeks of trauma, characterized by sharp margins and no periosteal reaction or callus formation [34]. Complete fracture was defined as corresponding inner and outer cortex disruption. An incomplete fracture was described as a disruption in the inner or outer cortex, such as a buckle rib fracture, characterized by a kink-like inner cortical disruption and a smooth outer cortex [35,36].

### Computed tomography and image processing

Images were acquired using a GE Optima CT660 scanner (GE Healthcare, Milwaukee, WI, USA) and a Philips Incisive CT scanner (Philips Healthcare, Cleveland, OH, USA). The standardized scanning parameters were as follows: tube voltage, 120 kV; tube current, automated dose modulation (1. ASIR, GE Optima CT660, and 2. iDose4, Philips Incisive CT); matrix size, 512 × 512; pitch, 0.516 (GE Optima CT660) or 0.85 (Philips). Bone reconstruction was performed on all images; the reconstruction slice thickness was 1.25 mm. The scanning range was from the root of the neck to the lower edge of the 12^th^ rib.

### CAD system

**Fig 2:**
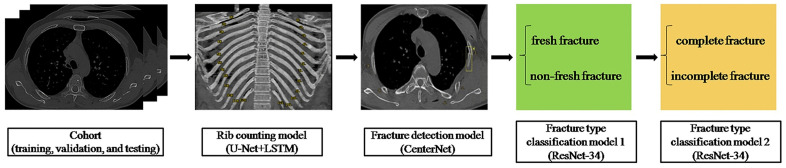
Brief diagram of the algorithm architecture. Model 1: ResNet-34 was used to distinguish a nonfresh fracture from a fresh fracture. Model 2: ResNet-34 was used to determine an incomplete fracture from a complete fracture among fresh fractures.

Our CAD system (Care.Ai Release 2023.1, Deep Wise Healthcare, Beijing, China) used the DL algorithm (S1 File) and included three main models: rib counting, fracture detection, and fracture type classification (Fig 2). No scans from the current dataset for the 8:1:1 training, validation, and testing of the DL algorithm were included. The diagnostic efficiency of the internal test dataset was 92.8% sensitivity for fresh rib fractures at the lesion level with a FPPP of 1.25. Furthermore, the model had a sensitivity of 85.1% in distinguishing between incomplete and complete fractures.

### Image interpretation

**Fig 3:**
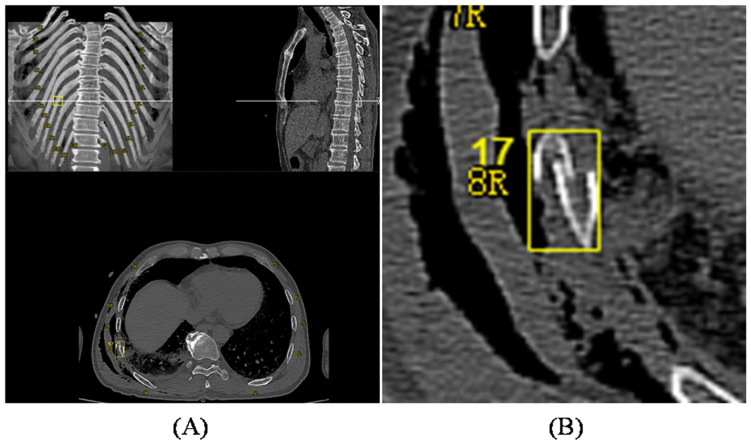
CAD system interface for automatic fracture identification and localization. **(A)** In the third round, each fresh fracture location was automatically identified and marked with a rectangular box by the CAD system. The fracture type was provided to the radiologist for reference. **(B)** The fracture location was partially magnified.

Six radiologists, each with 7 years of experience in thoracic CT diagnosis from three institutions, were blinded to the patient’s age, sex, and diagnostic efficiency of the CAD system in the internal test dataset. They used various viewing techniques—including axial view, multiplanar reconstruction, curved planar reconstruction, and volume rendering—to diagnose and categorize fresh rib fractures. They recorded the fresh rib fracture locations, types (complete/incomplete fractures), and reading time for each reading in a structured spreadsheet. Three rounds of reading were conducted (Fig 1). In the first round, to assess consistency in rib fracture diagnosis among the six radiologists, each radiologist independently read the CT images of all 1,012 patients. A 1-month washout interval was implemented between the rounds to minimize recall bias. Using R software (version 3.5.2), the 1,012 patients were then stratified by age (668 middle-aged/young vs. 344 older patients) and randomly allocated into six balanced subgroups (168–169 patients per group), with each maintaining the representative age ratio (approximately 1.94:1). In the second round, each of these six subgroups were assigned to each of the six radiologists for independent reading (defined as radiologist-only reading). In the third round, the radiologists were assigned subgroups identical to those in the second round and read the CT images jointly with the DL-based CAD system (defined as radiologist-CAD reading). In this round, each radiologist reviewed the CT images with each data point (location and fracture type) marked by the CAD system (Fig 3). Subsequently, they determine whether to accept or reject each data point. Following three rounds of reading, the reason for each misdiagnosis using the radiologist-only reading was determined by the same intermediate-level radiologist who performed the reading and by consultation with two senior radiologists.‌‌

### Reference standard for rib fractures

The initial and follow-up CT images were independently read by two senior radiologists, each with 15 years of experience in thoracic CT diagnosis, who represented the reference standard, including the locations and types of fresh rib fractures. The expert panel asked a thoracic surgeon to participate if the findings were ambiguous. The findings of the expert panel’s final discussions were used as reference standards [37].

### Statistical analysis

An independent data expert not involved in the readings counted the results in three rounds according to the reference standards. A fracture corresponding to the reference standard’s location and type was considered a true positive. A fracture that did not align with the reference standard in either location or type was considered a false positive. A fracture confirmed by the reference standard but regarded as normal was a false negative. Owing to the infinite number of normal positions in one rib, assessing the number of true negatives was impossible. Sensitivity was calculated by dividing the number of true positives by the overall number of fractures, as confirmed by an expert panel. In addition, the FPPP was calculated by dividing the number of false positives by the overall number of patients [26]. The primary endpoint was the sensitivity of radiologist-only reading for fresh fractures in the two different age groups and radiologist-CAD reading in the older group.

The R software version 3.5.2 (R Foundation for Statistical Computing, Vienna, Austria) was used to process the data. The χ^2^ test was used to compare the sex distribution, sensitivity, and FPPP between two age groups. McNemar’s test was used to compare the sensitivity and FPPP of the two reading methods. Kendall’s coefficient of concordance was used to assess the consistency of diagnostic findings among the six radiologists. An independent-sample *t*-test was used to compare reading time between the two age groups. A paired-sample *t*-test was used to compare the reading times of the two reading methods. Statistical significance was defined as a *p-*value of 0.05.

## Results

### Consistency in diagnosis of fresh fractures among six radiologists

Sensitivities for fresh fracture diagnosis among the six radiologists ranged from 87.9% to 88.2%. FPPPs for fresh fracture diagnosis among the six radiologists ranged from 0.42 to 0.44 (Table 1 in S2 File). Kendall’s W analysis revealed significant inter-observer consistency, with high agreement for sensitivities (W = 0.834, *p* < 0.001) and FPPPs (W = 0.855, *p* < 0.001).

### Number of true fractures in two different age groups

Each patient was represented by a single scan in this study. Overall, 1012 patients had 2,085 fresh fractures diagnosed per the reference standard (Table 2). Each of 163 patients had no fresh fracture. Each of 278 patients had one fresh fracture. Each of 571 patients had two or more fresh fractures.

**Table 2:** Number of true fractures per age group.

	Fresh fractures	Complete fractures	Incomplete fractures
**Group 1 (n = 668)**	1376	1017	359
**Group 2 (n = 344)**	709	528	181

**Note:** Group 1 and Group 2 represent the middle-aged/young group and the older group, respectively.

### Comparison of sensitivity using two reading methods in two different age groups

The sensitivity of radiologist-only reading method for fresh fractures in the older group was significantly lower than that of the middle-aged/young group (86.7% vs. 91.5%, *p* = 0.001). With CAD assistance, the sensitivity in the older group increased to 92.5% (*p* = 0.001), which did not differ significantly from the radiologist-only sensitivity in the middle-aged/young group (92.5% vs. 91.5%, *p* = 0.417; Table 3). The results of true positive detection in the two different age groups are shown in Tables 2 and 3 of S2 File.

**Table 3:** Comparison of sensitivity using two reading methods in two groups.

	Radiologist-only	Radiologist-CAD	χ^2^	*p* value
**Group1**	91.5% (1259/1376) (95% CI: 89.9%, 92.9%)	95.0% (1307/1376) (95% CI: 93.7%, 96.0%)	12.659	< 0.001
**Group2**	86.7% (615/709) (95% CI: 84.0%, 89.0%)	92.5% (656/709) * (95% CI: 90.4%, 94.2%)	12.094	0.001
χ^2^	11.632	5.143		
*p* value	0.001	0.023		

**Note:** Group 1 and Group 2 represent the middle-aged/young group and the older group, respectively. * The sensitivity for radiologist-CAD in the older group was not significantly different from that for radiologist-only reading in the middle-aged/young group (92.5% vs. 91.5%; χ^2^ = 0.660, *p* = 0.417). CI, confidence interval

### Comparison of sensitivity for diagnosing each fracture type using two reading methods in two different age groups

**Fig 4:**
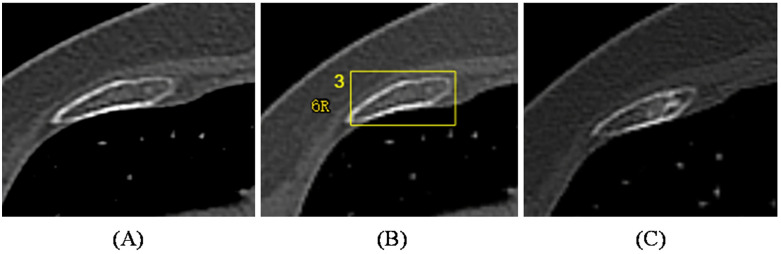
Representative case of incomplete rib fracture detection by the radiologist-CAD reading method. **(A)** A 63-year-old male with a car accident injury showed only a cortical fold of the bone in the right sixth anterior rib. **(B)** The incomplete fracture (rectangular box marked) was missed in the radiologist-only reading, whereas it was accurately diagnosed in the radiologist-CAD reading. **(C)** Four weeks later, a follow-up computed tomography review showed the formation of a callus at the location, confirmed as an incomplete fracture by the panel.

The sensitivities of radiologist-only method for complete and incomplete fractures in the older group were significantly lower than those in the middle-aged/young group (89.3% vs. 92.7%, *p* = 0.024 for complete fractures; 80.8% vs. 87.8%, *p* = 0.023 for incomplete fractures). With CAD assistance, the sensitivities in the older group increased to 93.5% (*p* = 0.003) and 90.6% (*p* = 0.001), respectively, which did not differ significantly from the sensitivities of radiologist-only method in the middle-aged/young group (93.5% vs. 92.7%, *p* = 0.558; 90.6% vs. 87.8%, *p* = 0.304, respectively; Table 4). The increase in sensitivity was greater for incomplete fractures than for complete fractures (9.8% vs. 4.2%, *p* = 0.004; Fig 4 and Table 4).‌‌

**Table 4:** Comparison of sensitivity of each fracture type using two reading methods in two groups.

		Radiologist-only	Radiologist-CAD	χ^2^	*p* value
**Group 1**	**Complete fractures**	92.7% (957/1032) (95% CI: 91.0%, 94.1%)	95.7% (988/1032) (95% CI: 94.3%, 96.8%)	21.356	< 0.001
**Group 2**	**Complete fractures**	89.3% (443/496) (95% CI: 86.3%, 91.7%)	93.5% (464/496) * (95% CI: 91.0%, 95.4%)	9.383	0.003
χ^2^		5.099	3.393		
*p*		0.024	0.065		
**Group 1**	**Incomplete fractures**	87.8% (302/344) (95% CI: 83.9%, 90.8%)	92.7% (319/344) (95% CI: 89.5%, 95.0%)	10.240	0.001
**Group 2**	**Incomplete fractures**	80.8% (172/213) (95% CI: 74.9%, 85.5%)	90.6% (193/213) ^▲^ (95% CI: 85.9%, 93.8%)	10.811	0.001
χ^2^		5.141	0.798		
*p* value		0.023	0.372		

**Note:** Group 1 and Group 2 represent the middle-aged/young group and the older group, respectively. * The sensitivity of complete fractures for radiologist-CAD in the older group (93.5%) was not significantly different from that for radiologist-only reading in the middle-aged/young group (92.7%) (χ^2^ = 0.342, *p* = 0.558). ^▲^ The sensitivity of incomplete fractures for radiologist-CAD in the older group (90.6%) was not significantly different from that for radiologist-only reading in the middle-aged/young group (87.8%) (χ^2^ = 1.057, *p* = 0.304). With CAD assistance, the increase in sensitivity was greater for incomplete fractures (9.8%) than for complete fractures (4.2%) (χ^2^ = 8.461, *p* = 0.004).

### Comparison of FPPP using two reading methods in two different age groups

**Fig 5:**
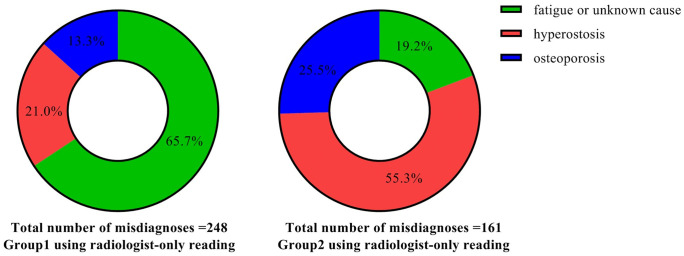
Reasons for misdiagnosis in Group1 and Group2 using radiologist-only reading, respectively. Group 1 and Group 2 represent the middle-aged/young group and the older group, respectively.

The FPPP of radiologist-only reading method for fresh fractures in the older group was significantly higher than that of the middle-aged/young group (0.47 vs. 0.37, *p* = 0.003). With CAD assistance, the FPPP in the older group decreased to 0.37 (*p* = 0.001), which did not differ significantly from the radiologist-only FPPP in the middle-aged/young group (0.37 vs. 0.37, *p* = 0.948; Table 5). The results of false positive detection in the two different age groups are shown in Tables 4 and 5 of S2 File. The reasons for misdiagnosis, including false negatives and false positives, with radiologist-only reading in the two different age groups are shown in Fig 5.

**Table 5:** Comparison of FPPP using two reading methods in two groups.

	Radiologist-only	Radiologist-CAD	χ^2^	*p* value
**Group 1**	0.37 (248/668)	0.39 (263/668)	1.573	0.242
**Group 2**	0.47 (161/344)	0.37 (127/344) *	12.042	0.001
χ^2^	8.829	0.577		
*p* value	0.003	0.448		

**Note:** Group 1 and Group 2 represent the middle-aged/young group and the older group, respectively. * The FPPP for radiologist-CAD in the older group was not significantly different from that for radiologist-only reading in the middle-aged/young group (χ^2^ = 0.004, *p* = 0.948).

### Comparison of the reading time using two reading methods in two different age groups

**Fig 6:**
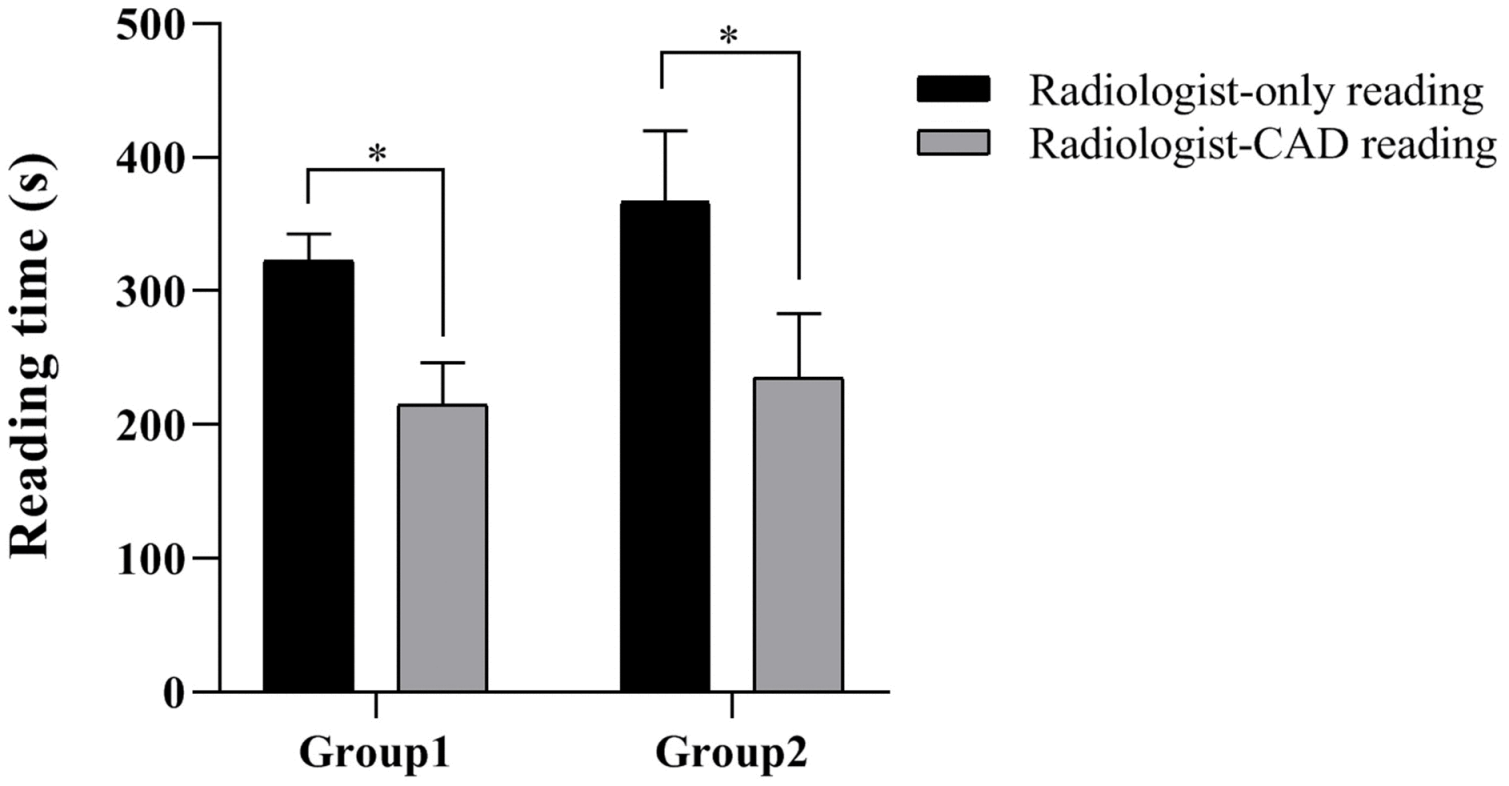
Reading time when using two reading methods in two different age groups. Group 1 and Group 2 represent the middle-aged/young group and the older group, respectively. * *p* < 0.05.

The reading time of radiologist-only method for fresh fractures in the older group was significantly longer than that in the middle-aged/young group (6.1 min vs. 5.4 min, *p* < 0.001). With CAD assistance, the reading time in the older group decreased significantly compared with that of radiologist-only reading (3.9 min vs. 6.1 min, *p* < 0.001), corresponding to a reduction of approximately 36% (Table 6 and Fig 6).

**Table 6:** Comparison of the reading time (min) using two reading methods in two groups.

	Radiologist-only	Radiologist-CAD	t	*p* value
**Group 1**	5.4 ± 0.3	3.6 ± 0.5	78.401	< 0.001
**Group 2**	6.1 ± 0.9	3.9 ± 0.8	33.864	< 0.001
t	−21.947	−10.491		
*p* value	< 0.001	< 0.001		

**Note:** Group 1 and Group 2 represent the middle-aged/young group and the older group, respectively.

## Discussion

Recently, DL-based CAD systems have been used to diagnose rib fractures. Meng et al. [25] detected and classified rib fractures in 300 CT images and found that radiologists assisted by a DL model obtained a higher F1-score (0.960) and recall rate (0.978) than those who read independently. Kaiume et al. [26] stated that their DL-based CAD system for CT diagnosis yielded a sensitivity of 64.5% for identifying rib fractures, which was higher than reading the two interns alone. However, CT-based rib fracture diagnosis in older emergency patients has certain limitations. The CT imaging manifestations in older patients, which may include thinning of the rib cortex, reduction of bone trabeculae, rib cortical thickening or irregular morphology, due to the higher proportion of osteoporosis [6–8] and hyperostosis [17,18] in this age group, may hamper radiologists’ diagnosis of rib fractures and lead to a higher rate of misdiagnosis. In this study, using the radiologist-only reading, misdiagnosis occurred in both middle-aged/young and older groups. For instance, some false positives due to anatomical variations (e.g., costochondral junctions) could be mistaken for fractures. Additionally, minor, nondisplaced fractures in areas of high complexity, such as the posterior ribs, could be missed as false negatives. Therefore, we used a DL-based CAD system for the diagnosis of rib fractures. We found that the fresh rib fracture diagnostic efficiency in the older group using the radiologist-only reading was lower than that in the middle-aged/young group and that with CAD assistance, the diagnostic efficiency could be optimized to the same level as that achieved in the middle-aged/young group when using the radiologist-only reading, with reduced reading time.

Our study indicated that radiologist-only reading for fresh fractures yielded lower sensitivity in the older group than in the middle-aged/young group. This may be due to the imaging manifestation characteristics of CT in the older group due to osteoporosis and hyperostosis, which may have interfered with the radiologists’ diagnosis of rib fractures. Furthermore, in recent years, some reports have indicated that the diagnostic efficiency of CT for rib fractures can be improved when CAD systems assist radiologists. For example, Jin et al. [24] developed a DL-based CAD system for detecting rib fractures in a test cohort of 120 patients. They achieved a higher detection sensitivity (93.4% and 94.4% for two radiologists, respectively) when using a collaborative human-computer reading approach than when using a computer or human reading only. Zhang et al. [27] indicated that using a DL model to assist two radiologists in diagnosing rib fractures in 198 chest CT images achieved higher sensitivity (88.9% and 88.7% for the two radiologists, respectively) than when the radiologists read independently. However, these studies have yet to consider the potential impact of age on fracture diagnostic efficiency or explore the value of CAD in diagnosing rib fractures in different age groups. We utilized the CAD system in two different age groups. We found that the sensitivity of diagnosis of fresh fractures when using the radiologist-CAD reading in the older group could be improved to the same level as that of the radiologist-only reading in the middle-aged/young groups. This suggests that using the DL-based rib fracture CAD system to assist radiologists in reading CT images of older patients may help reduce missed diagnoses of fresh fractures.

For each fracture type, this study indicated that using radiologist-only reading for incomplete and complete fractures in the older group exhibited lower sensitivity than in the middle-aged/young group. This suggests that the reduction in fresh rib fracture diagnostic sensitivity when using radiologist-only reading in the older group may be specific to missed diagnoses of incomplete or complete fractures. Furthermore, Zhou et al. [28] indicated that rib fractures could be classified into three categories (fresh, healing, and old). They reported that DL model II had a higher sensitivity for diagnosing fresh and healing fractures (89.6% and 97.6%, respectively) than five radiologists with 7–9 years of CT diagnostic experience. Inspired by their report, we used the CAD system in two age groups. We classified fresh fractures in emergency patients with blunt chest trauma that occurred within 3 weeks into incomplete and complete fractures. Our results indicate that with the assistance of the CAD system, the sensitivity of both incomplete and complete fractures in the older group increased to the same level as the radiologist-only or radiologist-CAD reading in the middle-aged/young group, in which the increase in incomplete fractures was 9.8%, which was higher than the 4.2% increase in complete fractures. Using the DL-based rib fracture CAD system to assist radiologists in reading CT images in an older patient group could help reduce missed diagnoses of incomplete and complete fractures. It may be beneficial in decreasing missed diagnoses of incomplete fractures.

Regarding FPPP, this study indicated that using radiologist-only reading to detect fresh fractures in the older group exhibited a higher FPPP than the middle-aged/young group. Zhang et al. [27] indicated that using a DL model to assist two radiologists in diagnosing rib fractures in 198 chest CT images achieved a lower false positives per scan of 0.13 for one of the radiologists than when the radiologists read independently. Castro–Zunti et al. [29] found that a DL model exhibited fewer false positive diagnoses than radiologists alone in detecting old fractures and normal ribs. We utilized the CAD system in two different age groups, and the results indicated that, with the assistance of the CAD system, the FPPP of fresh fractures in the older group decreased to the same level as that achieved with radiologist-only or radiologist-CAD reading in the middle-aged/young group. This suggests that the CAD system can help radiologists reduce the false positives of fresh fractures in older patients.

Regarding reading time, Liu et al. [38] reported that junior radiologists could reduce their reading time by 28% with the assistance of DL-based rib fracture artificial intelligence software. Sun et al. [39] reported that radiologists with less experience could reduce their reading time by 47%. In contrast, senior radiologists could reduce their reading time by 9% when assisted by a DL-based rib fracture CAD system. The results of our study are generally consistent with these reports, showing that radiologists’ reading time for images of an older patient group was longer than that of the middle-aged/young group. Moreover, we showed that CAD could assist radiologists in reducing the reading time in the older group by approximately 36%.

The CAD system has important implications when utilized in emergency settings, where radiologists are often overloaded with trauma cases. The CAD system can serve as a second reader, reducing missed diagnoses and false positives, and improving the speed of patient care, especially for older high-risk patients. Saved time with faster diagnosis of rib fractures may lead to quicker intervention, reducing complications such as pneumonia or respiratory failure, and potentially improving the prognosis of emergency patients.

However, to ensure a balanced discussion, it is important to acknowledge that CAD may mislead radiologists in certain scenarios. As shown in Tables 2–5 of S2 File, CAD may mislead radiologists, resulting in both false positives (misclassifying nonfracture structures) and false negatives (overlooking fractures). These errors may stem from model “hallucinations” or training data biases, highlighting the need for radiologists to maintain critical thinking and utilize CAD as an auxiliary tool rather than as definitive diagnostic basis.

Currently, the development of the CAD system faces several challenges, including ethical concerns, complex rib anatomy, the subtle nature of some fractures, and the need for high-quality imaging data. Variability in CT scan protocols and patient positioning can affect the accuracy of fracture detection. The CAD system should be validated on a diverse dataset to ensure its generalizability to different patient populations. Despite the challenges, we hypothesize that advanced CAD systems can accurately diagnose not only rib fractures but also fractures in other parts of the thorax (e.g., thoracic vertebral fractures) and incorporate clinical data such as patient age, history of osteoporosis, and other comorbidities to provide a comprehensive risk assessment. Also, further development of the CAD system can be done to recognize fracture healing stages and assess the potential for complications (e.g., rib displacement affecting lung function).

This study has some limitations. First, the six intermediate-level radiologists focused solely on diagnosing rib fractures, which is inconsistent with clinical practice. Second, we only included six radiologists with the same experience level, each possessing 7 years of experience, as readers to diagnose rib fractures from CT images. Further increase of radiologist number and inclusion of those with different experience levels would contribute to richer and more objective results. Third, it was difficult to quantify the contribution of the cumulative learning experience from repeated readings.

## Conclusions

In summary, intermediate-level radiologists’ efficiency in diagnosing fresh rib fractures by radiologist-only reading was lower in older emergency patients than in middle-aged/young patients. Moreover, the potential value of using a DL-based CAD system to assist radiologists in diagnosing rib fractures in older emergency patients may be higher than in middle-aged/young patients.

## Supporting information

S1 File Deep learning algorithm. (DOCX)

S2 File Sensitivity and false positives per patient (FPPP) of six radiologists for fresh fracture diagnosis, and true positive and false positive detection results in the middle-aged/young and older groups. (DOCX)
