# Supplementary material for: Computer-aided diagnosis system for thoracic computed tomography of rib fractures in older emergency patients: A preliminary study
Source: PLoS One. 2026 Jun 17;21(6):e0351988. doi: 10.1371/journal.pone.0351988 (PMC13274818; doi:10.1371/journal.pone.0351988)
Supplement: S1 File — (DOCX) [file pone.0351988.s001.docx]

**Supplementary Material 1**

**Deep learning algorithm**

The deep learning (DL) algorithm comprised three main models: a rib-counting model, a fracture-detection model, and a fracture-type classification model. The rib-counting model consisted of rib segmentation and counting subprocesses. The rib segmentation model utilized U-Net [1], which was based on a bidirectional long short-term memory (LSTM) framework, to segment rib regions; the LSTM modeled three-dimensional spatial connections. These two subprocesses were jointly trained to achieve higher segmentation accuracy. During the counting process, three-dimensional distances were calculated based on the segmentation results. The rib regions were refined using local geometric features. Dynamic programming was employed to optimize the counting cost function, separating bonded ribs and combining unconnected sections.

The fracture-detection model comprised an abnormality-screening module and a false-positive-elimination module arranged in a cascade framework. The abnormality-screening model, based on CenterNet [2], identified anomalous spatial locations. The false-positive-elimination model subsequently distinguished true fractures from false positives.

Additionally, rib fractures were classified into two types using a two-step approach. First, a model distinguished fresh fractures from non-fresh fractures. Second, a sub-model classified fresh fractures as either incomplete or complete. The classification models utilized a 34-layer residual network (ResNet-34) [3] with parameters fixed after pre-training on the ImageNet dataset.

A total of 10,847 consecutive CT scans performed between April 1, 2012, and November 30, 2022, at five hospitals were divided into training, validation, and testing sets at a ratio of 8:1:1. The model was developed using the PyTorch framework and trained on eight NVIDIA GeForce RTX 2080 Ti graphics processing units. On the internal test dataset, the model achieved a sensitivity of 92.8% for fresh rib fractures at the per-lesion level, with 1.25 false positives per patient. Furthermore, the model demonstrated a sensitivity of 85.1% for distinguishing incomplete from complete fractures.

**References**

1. Ronneberger O, Fischer P, Brox T, editors. U-net: Convolutional networks for biomedical image segmentation. Medical Image Computing and Computer-Assisted Intervention–MICCAI 2015: 18th International Conference, Munich, Germany, October 5-9, 2015, Proceedings, Part III 18; 2015: Springer.
2. Duan K, Bai S, Xie L, Qi H, Huang Q, Tian Q, editors. Centernet: Keypoint triplets for object detection. Proceedings of the IEEE/CVF international conference on computer vision; 2019.
3. He K, Zhang X, Ren S, Sun J, editors. Deep residual learning for image recognition. Proceedings of the IEEE conference on computer vision and pattern recognition; 2016.
