# Supplementary material for: Computer-aided diagnosis system for thoracic computed tomography of rib fractures in older emergency patients: A preliminary study
Source: PLoS One. 2026 Jun 17;21(6):e0351988. doi: 10.1371/journal.pone.0351988 (PMC13274818; doi:10.1371/journal.pone.0351988)
Supplement: S2 File — (DOCX) [file pone.0351988.s002.docx]

**Supplementary Material 2**

**1. The results of sensitivity and FPPP of six radiologists** **for fresh fracture diagnosis**

**Table 1. Sensitivity and FPPP of six radiologists** **for fresh fracture diagnosis**

|  | **Sensitivity** | **FPPP** |
| --- | --- | --- |
| **R1** | 87.9% (1833/2085)  (95% CI: **86.4%**, **89.2%**) | 0.42 (425/1012) |
| **R2** | 88.1% (1837/2085)  (95% CI: **86.6%, 89.4%**) | 0.43 (436/1012) |
| **R3** | 88.0% (1835/2085)  (95% CI: 86.5%, 89.3%) | 0.43 (436/1012) |
| **R4** | 88.1% (1837/2085)  (95% CI: **86.6%, 89.4%**) | 0.44 (445/1012) |
| **R5** | 87.9% (1833/2085)  (95% CI: **86.4%**, **89.2%**) | 0.43 (436/1012) |
| **R6** | 88.2% (1839/2085)  (95% CI: **86.7%, 89.5%**) | 0.44 (445/1012) |

**Note:** FPPP**,** false positives per patient; R1, R2, R3, R4, R5, and R6 represent radiologists 1, 2, 3, 4, 5, and 6, respectively.

**2. The results of true positive detection in the middle-aged/young group**

**Table 2. True positive (lesions/patients) detection in the middle-aged/young group**

| **Reading method** | | **Radiologist-only** | |
| --- | --- | --- | --- |
|  |  | **detected** | **undetected** |
| **Radiologist-CAD** | **detected** | 1192/517 | 115/49 |
|  | **undetected** | 67/33 | 2/2 |

**Note:** 67 patients had no true positive fractures according to the reference standard.

**3. The results of true positive detection in the older group**

**Table 3. True positive (lesions/patients) detection in the older group**

| **Reading method** | | **Radiologist-only** | |
| --- | --- | --- | --- |
|  |  | **detected** | **undetected** |
| **Radiologist-CAD** | **detected** | 566/235 | 90/39 |
|  | **undetected** | 49/24 | 4/3 |

**Note:** 43 patients had no true positive fractures according to the reference standard.

**4. The results of false positive detection in the middle-aged/young group**

**Table 4. False positive (lesions/patients) detection in the middle-aged/young group**

| **Reading method** | | **Radiologist-only** | |
| --- | --- | --- | --- |
|  |  | **detected** | **undetected** |
| **Radiologist-CAD** | **detected** | 184/178 | 79/75 |
|  | **undetected** | 64/62 | 0/367 |

**N****ote:** Six patients had fractures classified as false positives by both radiologist-only and radiologist-CAD, as well as additional fractures classified only by radiologist-only; eight patients had fractures classified by both, as well as additional fractures classified only by radiologist-CAD.

**5. The results of false positive detection in the older group**

**Table 5. False positive (lesions/patients) detection in the older group**

| **Reading method** | | **Radiologist-only** | |
| --- | --- | --- | --- |
|  |  | **detected** | **undetected** |
| **Radiologist-CAD** | **detected** | 96/91 | 31/30 |
|  | **undetected** | 65/62 | 0/174 |

**Note:** Seven patients had fractures classified as false positives by both radiologist-only and radiologist-CAD, as well as additional fractures classified only by radiologist-only; six patients had fractures classified by both, as well as additional fractures classified only by radiologist-CAD.
